# Giant cutaneous horn in an African American woman: Clinical observations and population insights

**DOI:** 10.1016/j.jdcr.2024.11.011

**Published:** 2024-11-25

**Authors:** Vixey Silva, Bret-Ashleigh Coleman, Heather Kopecky, Richard Miller, Keith Baribault

**Affiliations:** a Department of Dermatology, HCA Healthcare/USF Morsani College of Medicine GME: HCA Florida Largo Hospital, Largo, Florida; b Edward Via College of Osteopathic Medicine, Auburn, Alabama; c Dermpath Diagnostics, Tampa, Florida

**Keywords:** actinic keratosis, African American, cutaneous horn, giant cutaneous horn, verruca vulgaris

## Introduction

A cutaneous horn (CH) is a hyperkeratotic, conical projection resembling an animal horn. When it exceeds 1 cm in height, it is classified as a giant cutaneous horn (GCH). The CH itself is not a pathological diagnosis but arises from an underlying lesion identifiable through histological examination. More than half of CHs are associated with premalignant or malignant causes, with actinic keratosis and squamous cell carcinoma (SCC) being the most frequent, respectively.^1^ In contrast, GCHs are often linked to benign conditions like verruca vulgaris, although SCC and sebaceous carcinoma have been reported.^2^ GCHs are more commonly reported in Caucasian and Asian populations and are rare among African Americans. These lesions typically occur in areas exposed to UV radiation or burns, with the highest frequency on the head, neck, and face. This case report details the uncommon occurrence of a GCH in an African American woman and is the first to provide an overview of GCHs in the African American population.

## Case report

**Fig 1:**
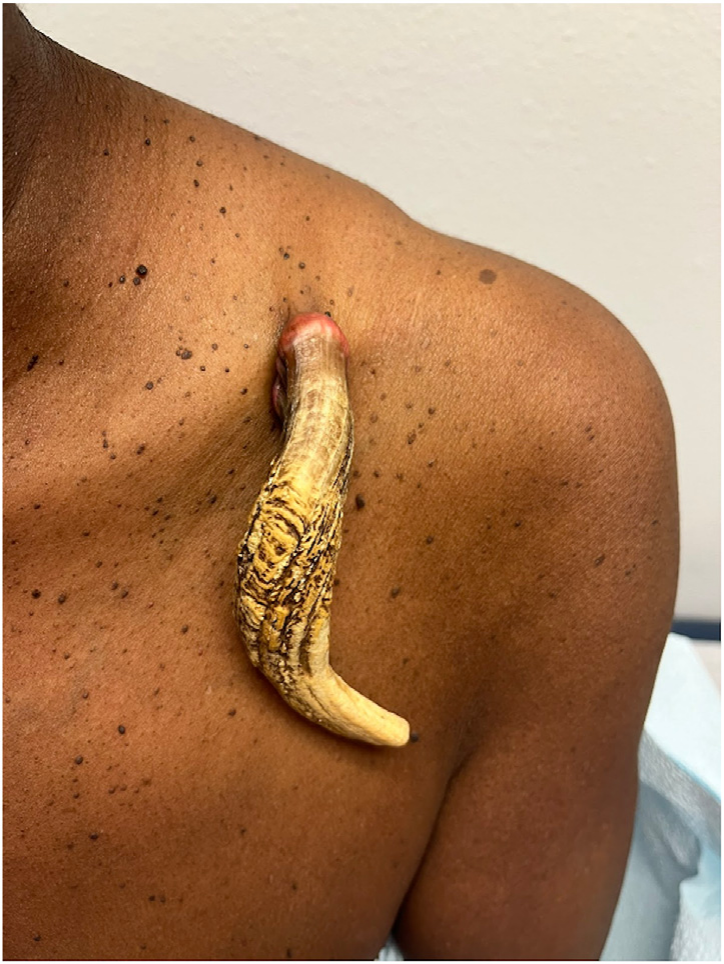
Conical, curving, hyperkeratotic horn with an erythematous base arising the left lateral clavicle.

A 61-year-old African American female with no significant past medical history presented to the clinic for removal of a hyperkeratotic growth that had been enlarging for 6 years. Physical examination revealed a 12.5 cm long, conical, curving, hyperkeratotic horn protruding from her left lateral clavicle (Fig 1). The base of the lesion measured 2 cm in diameter with a smooth erythematous appearance and was hanging off a flesh-colored stalk. The patient reported associated pain with manipulation and irritation from clothing, which affected her daily activities of living. Due to lack of health insurance, she had delayed seeking earlier treatment.

**Fig 2:**
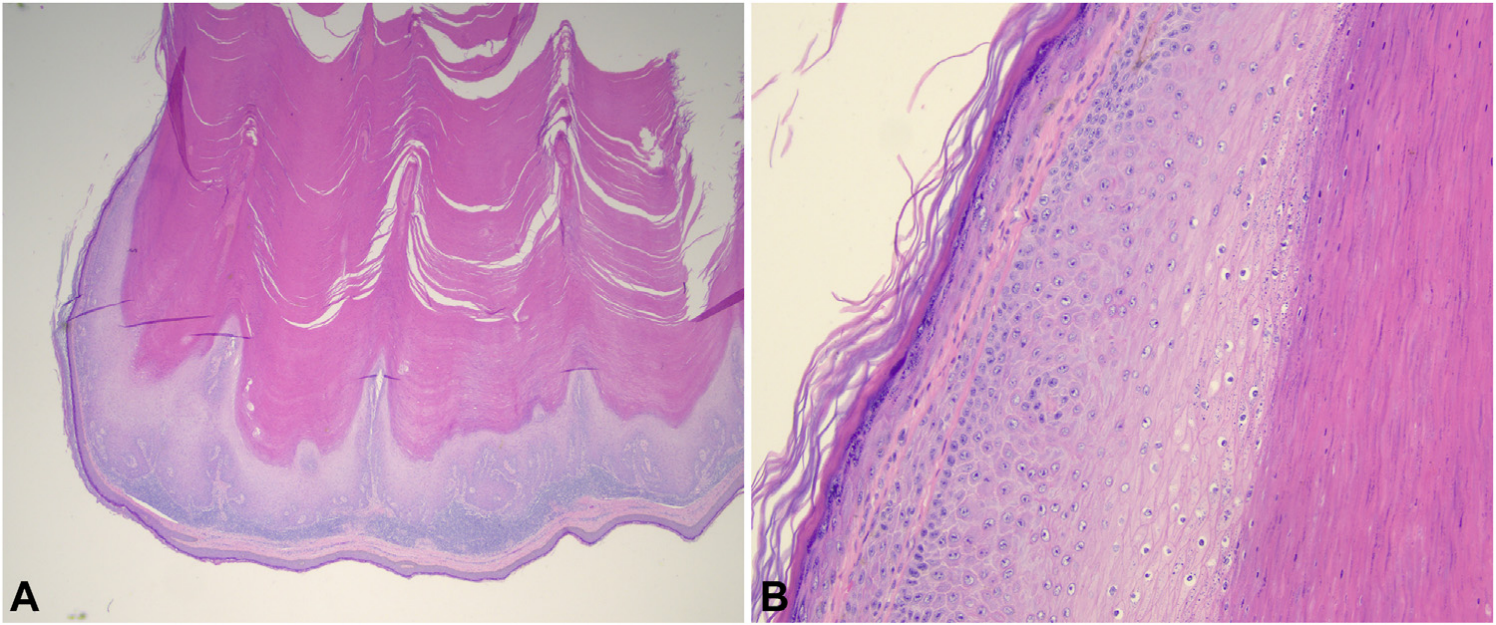
**A,** Hematoxylin and eosin staining at 20× magnification revealing verrucous acanthosis, invaginated hyperkeratosis, and a lymphocytic infiltrate within the superficial dermis, consistent with an inflamed verruca. **B,** Hematoxylin and eosin staining at 100× magnification shows the epidermis without cytologic atypia.

A broad deep shave technique was employed for removal, and the wound was left to heal by secondary intention. A 1 cm long piece from the proximal part of the horn was separated using extra-large nail nippers and sent for pathological examination. Histology revealed verrucous acanthosis, invaginated hyperkeratosis without epithelial atypia, and a lymphocytic infiltrate within the superficial dermis consistent with an inflamed verruca (Fig 2, *A* and *B*). The patient's biopsy site healed without complications, and no recurrence was observed during a 5-month follow-up period.

## Discussion

The most commonly reported lesion at the base of GCHs is verruca vulgaris, although SCC and sebaceous carcinoma have also been documented. A literature review conducted by the authors on PubMed in August 2024 identified 6 reported cases of GCH in the African American population, focusing only on manuscripts specifying patients of African American descent, as shown in Table I. None of these cases involved premalignant or malignant bases. Benign verrucous lesions like verruca vulgaris and verrucoid-like hyperplasia were the most frequently reported, as seen in our case. Other lesions included epidermal nevus and nonspecific descriptions characterized by epidermal acanthosis and hyperkeratosis, both benign entities without evidence of malignancy. The absence of sun-related premalignant and malignant lesions, such as actinic keratosis and SCC, at the base of GCHs in African Americans is due to increased melanin, which absorbs ultraviolet rays, neutralizes free radicals, and protects keratinocytes from damage. Despite the lower incidence of carcinomas associated with GCHs in the African American population, it is important to biopsy and remove these lesions due to cosmetic concerns and the potential risk of malignancy, as observed in the general population.

**Table I:** Cases of giant cutaneous horns in the African American population and their characteristics

Study	Age	Sex	Site	Histological diagnosis	Length size
Semins et al (1969)^3^	69	M	Arm	Mild papillary hypertrophy with overlying hyperparakeratosis	7.1 cm
Nthumba et al (2007)^4^	28	F	Scalp	Verrucoid epidermal hyperplasia	3 cm
Sutphin et al (2012)^5^	38	F	Scalp	Acanthomatous hyperplasia and keratin deposition	8 cm
Leppard et al (2014)^6^	52	F	Scalp	Verruca vulgaris	12 cm
Cardis et al (2017)^7^	10	M	Neck	Epidermal nevus	2.5 cm
Nussbaum et al (2019)^1^	65	F	Back	Verruca vulgaris	22 cm

*F*, Female; *M*, male.

Associated pain and erythema at the base are significant indicators of an increased risk of malignancy, as are lesions with a wide base or a low height-to-base ratio, which are more likely to be premalignant or malignant.^8^ GCHs that are longer in length have been documented to arise from preexisting verruca, as showcased by the longest documented case of GCH at 22 cm, as seen in Table I. In addition to specific lesion characteristics indicating a higher likelihood of malignancy, factors such as race, sex, and age can also suggest malignant potential, with Caucasians, advanced age, and male sex associated with a higher risk of malignancy and therefore warranting close monitoring.

While primarily found in adults in the sixth and seventh decades of life, there are few reports of GCHs in younger adults and children. GCHs found in the younger population were more likely to have benign base lesions such as epidermal nevi and pyogenic granulomas.^7^ We identified only 1 pediatric case of GCH in a 10-year-old African American child with an underlying epidermal nevus. The rarity of GCH in children is likely due to decreased cumulative sun exposure and parents seeking health care evaluation more readily for smaller lesions. In contrast, adults may defer treatment due to factors such as insurance concerns and fear, despite experiencing similar pain and disfigurement. This disparity in health care-seeking behavior may explain the large variation in GCH sizes among the population and the predilection for larger lesions in older individuals.

Due to the risk of malignancy, it is crucial to exercise extreme caution when excising these lesions, ensuring that the entire base is removed for histological examination to rule out further malignancy, preferably with a deep broad shave, as detailed in our case. Currently, there are no specific treatment guidelines for CHs with underlying malignancy; thus, treatment should follow the guidelines for the underlying malignancy. Patients with CHs associated with underlying SCC should be evaluated for metastasis and monitored closely for the first 3 years after diagnosis to detect any rare metastasis.^9^ While there have been no reported cases of metastatic SCC under a CH or GCH, 3 cases of malignant melanoma under a CH have been documented.^2^ Therefore, vigilant monitoring and appropriate treatment are essential to manage these lesions effectively.

## Conflicts of interest

None disclosed.
